# The Human Milk Microbiome in Mothers of Very-Low-Birth-Weight Infants: A Systematic Review of Recent Clinical Studies

**DOI:** 10.3390/children13060790

**Published:** 2026-06-06

**Authors:** Vilma Ivanauskienė, Aušrelė Kudrevičienė, Vaida Aleksejūnė, Renata Dzikienė, Ilona Aldakauskienė, Rasa Tamelienė

**Affiliations:** Department of Neonatology, Faculty of Medicine, Lithuanian University of Health Sciences, LT-44307 Kaunas, Lithuania; ausrele.kudreviciene@lsmu.lt (A.K.); vaida.aleksejune@kaunoklinikos.lt (V.A.); renata.dzikiene@lsmu.lt (R.D.); ilona.aldakauskiene@lsmu.lt (I.A.)

**Keywords:** preterm newborn, breast milk, human milk, microbiota, microbiome, sequencing techniques

## Abstract

**Highlights:**

**What are the main findings?**

• Mother’s own milk (MOM) of very-low-birth-weight (VLBW) (<1500 g) preterm infants contains a distinct and changing microbiota dominated by *Staphylococcus*, *Streptococcus*, and *Enterococcus*.
• Milk microbiota diversity increases during lactation and influences infant gut colonization.

**What are the implications of the main findings?**

• MOM supports early immune and gut microbiome development in VLBW infants.
• Further multi-omic and longitudinal studies are needed to clarify clinical effects and long-term outcomes.

**Abstract:**

Preterm birth remains a major global health concern, affecting approximately one in ten neonates, with an estimated 15 million infants born prematurely each year. Prematurity and clinical factors such as antibiotics, cesarean delivery, and limited access to mother’s own milk disrupt microbiota development in VLBW infants; although human milk supplies nutrients and a microbial community, its composition and clinical role are not yet well understood. However, the composition and clinical significance of the human milk microbiota (HMM) in VLBW infants remain insufficiently characterized. Background: This review aims to summarize recent evidence (2021–2025) on the microbiome of MOM in mothers of VLBW (<1500 g) preterm infants and to evaluate its potential role in neonatal health. Methods: The study used a systematic literature review, searching PubMed and Google Scholar with predefined criteria and keywords. Results and Conclusions: MOM microbiota of VLBW in infants is dominated by *Staphylococcus*, *Enterococcus*, *Streptococcus*, *Enterobacteriaceae*, and *Acinetobacter*, with lower levels of *Veillonella*, *Clostridium sensu stricto*, *Pseudomonas*, *Haemophilus*, and *Bifidobacterium*; its diversity increases over lactation, and feeding type influences infant gut colonization and immune development, though links to necrotising enterocolitis (NEC) remain limited. Further research using multi-omic approaches is needed to clarify these mechanisms and their clinical implications.

## 1. Introduction

The human microbiota consists of all microorganisms living in the body. The Human Microbiome Project (2008) was launched to study how these microbes, their genomes, and environments affect health and disease. 16S rRNA sequencing has enabled detailed analysis of microbial communities, helping to define a “healthy” microbiota and understand its role in disease [1,2].

Preterm birth is a non-physiological condition linked to early clinical factors that may disrupt normal microbial colonization [3,4]. For infants born prematurely, early microbial disruption may influence how the immune system develops well beyond infancy [5,6]. Breast milk is vital for premature infants, providing nutrients that support intestinal development and maturation. In both term and preterm infants, MOM may also supply bacteria that help colonize the gut [6,7].

The gut microbiota of preterm infants differs significantly from that of term infants and is influenced by gestational age, delivery mode, antibiotics, and diet. Breast milk provides the most suitable nourishment for infants, supporting their growth and developmental processes. It also contains bioactive components such as beneficial microbes, human milk oligosaccharides, immunoglobulins, lactoferrin, and polyunsaturated fatty acids, which support immune development. These components interact with gut bacteria and immune cells, promoting microbiome establishment, regulating inflammation, and supporting gut health. Breast milk bacteria further help shape microbial composition and inflammatory responses [8,9,10,11,12,13]. The maternal gut microbiome is considered an important contributor through the proposed entero-mammary pathway, involving transfer of bacteria or bacterial components from the maternal intestine to the mammary gland [14].

**Figure 1:**
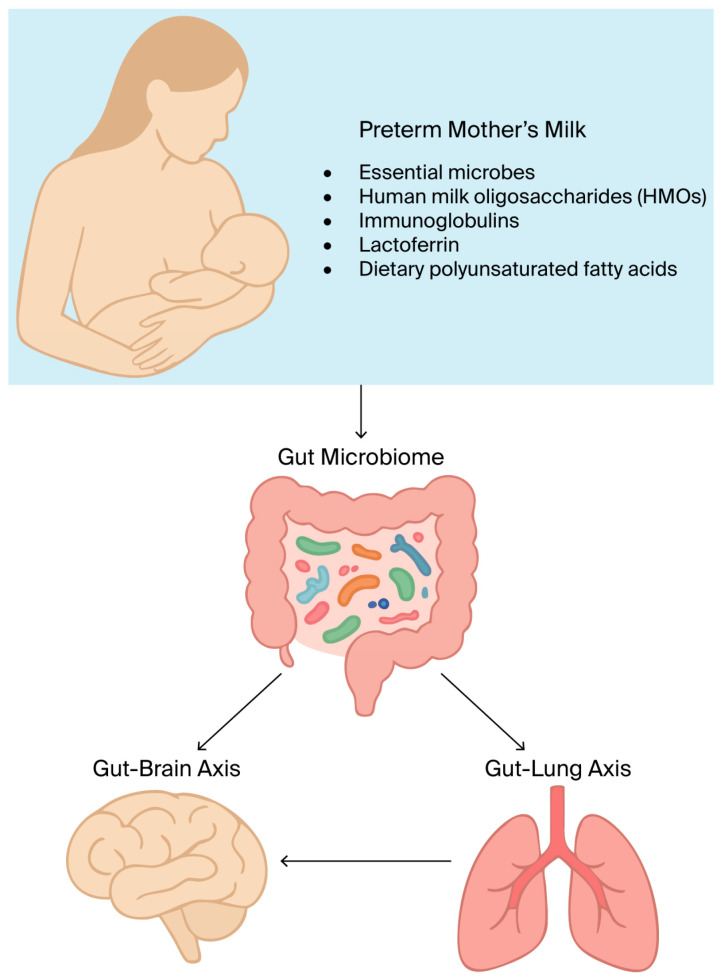
Schematic representation created using OpenAI’s ChatGPT (GPT-5, October 2025), illustrating the relationships between bioactive components of preterm mother’s milk, the gut microbiome, and the gut–brain–lung axes. The figure depicts how essential microbes, human milk oligosaccharides (HMOs), immunoglobulins, lactoferrin, and polyunsaturated fatty acids in preterm milk contribute to gut microbiome establishment, which in turn communicates bidirectionally with the central nervous system (CNS) and influences the lung microbiome. Together, these interconnected pathways regulate immunity, neurodevelopment, and respiratory health in preterm infants.

Intestinal dysbiosis in preterm infants is increasingly studied due to its impact on health. Evidence suggests gut–lung and gut–brain axes link intestinal microbes with immune and neurodevelopmental outcomes (Figure 1). Disrupted gut–brain communication has been associated with neurological disorders, including autism spectrum disorder, multiple sclerosis, and Parkinson’s disease [2,15,16,17].

Human breast milk has its own microbiome and is a key source of bacteria for infant gut colonization. Its microbes may promote anti-inflammatory responses, help protect against infections, and include strains with probiotic potential. MOM or donor milk is preferred for preterm infants, though fortification is often needed, and formulas may be used when milk is insufficient. However, the effects of different feeding practices on the preterm gut microbiome are still not well understood. Overall, breast milk microbes may support gut colonization and immune development, but limited research has been conducted on this topic [18,19].

This study aims to systematically review and synthesize current evidence on the microbiota of MOM in VLBW preterm infants, with a focus on its composition, changes throughout lactation, and its association with infant morbidity outcomes.

We specifically aimed to address the following questions:

• What is currently known about the MOM microbiota in mothers of VLBW preterm infants?
• How does the MOM microbiota in mothers of VLBW preterm newborns change over the course of lactation?
• What is known about the association between MOM microbiota and morbidity outcomes in VLBW infants?

## 2. Materials and Methods

### 2.1. Study Design

This systematic review was conducted and reported in accordance with the Preferred Reporting Items for Systematic reviews and Meta-Analyses (PRISMA) guidelines [20] (Supplementary Material Table S1).

### 2.2. Literature Search

The literature review was conducted using the PubMed and Google Scholar databases, as well as the databases accessible through the Lithuanian University of Health Sciences (last accessed on 16 June 2025), in addition to hand searches for primary studies investigating the microbiota of mothers’ own milk in VLBW (<1500 g) preterm newborns. Relevant studies were identified through a literature search using the following keywords, used individually or in various combinations: “human milk” OR “breast milk” AND “preterm newborn” OR “preterm neonate” OR “preterm infants” AND “microbiota” OR “microbiome” AND “sequencing techniques” OR “sequencing methods”. The inclusion criteria were as follows: classical article, clinical study, clinical trial, controlled clinical trial, case reports, corrected and republished articles published in PubMed and Google Scholar databases, and studies using humans. Studies published in languages other than English, lacking accessible full-text versions, or classified as grey literature [21] (conference abstracts, theses, governmental or organizational reports, and other unpublished materials) were excluded. Only studies available as full-text publications in peer-reviewed scientific journals were considered eligible to ensure methodological quality and data reliability. To supplement the database search, cited references within the initially included articles were manually reviewed for additional studies [21].

Before initiating the literature search, each author established a personal account within the selected databases, allowing search results to be stored and retained for future reference. A shared Excel file was then developed to document key study characteristics, including publication year, author names, and article titles. This file served as a screening and tracking tool throughout the study selection process [21]. Studies focusing exclusively on the gut microbiome of preterm infants were excluded, as the review was specifically designed to investigate the microbiome of MOM in VLBW infants.

### 2.3. Eligibility Criteria

Studies were included if they were:

1. Case–control and cohort studies analyzing the MOM microbiota of mothers who delivered VLBW preterm infants.
2. Case–control and cohort studies investigating changes in the MOM microbiota of VLBW preterm infants’ mothers during the course of lactation.
3. Case–control and cohort studies investigating the association between the MOM microbiota and morbidity and long-term outcomes in preterm infants.

### 2.4. Quality Assessment

The quality of the studies was assessed by two researchers according to the following criteria (Table 1), using the Newcastle–Ottawa Scale for Assessing the Quality [22].

The components used to calculate the overall score are categorized into four domains [21,22]:

1. Clearness of the aim (maximum 2 points);
2. Sample selection (maximum 8 points);
3. Comparability (maximum 2 points);
4. Outcome (maximum 4 points).

Total score [21,22]:

13–16 points: high quality and low risk of bias;

9–12 points: moderate quality and moderate risk of bias;

Less than 9 points: low quality and high risk of bias.

## 3. Results

### 3.1. Study Results

**Figure 2:**
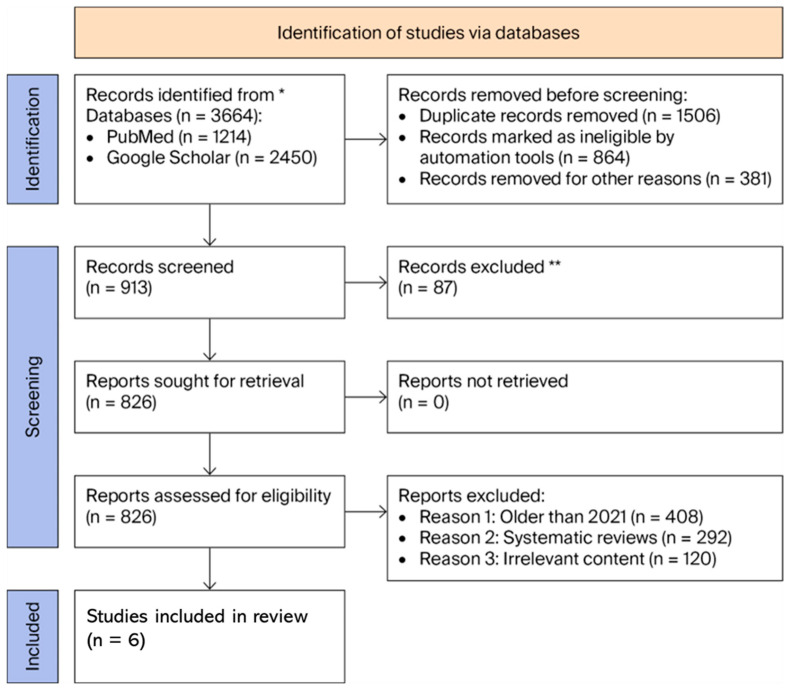
Identification of studies via databases (PRISMA flow diagram) [23]. * Records identified through database searching conducted for this systematic review. ** Not related to the review topic, not human studies, duplicate records not identified earlier.

A total of 3664 articles were obtained through searches of PubMed and Google Scholar. After screening titles and abstracts, 913 studies remained, excluding those with only abstracts available or published before 2021. Full-text assessment further excluded studies due to duplication, systematic reviews, or irrelevance to the topic, leaving 826 articles. Ultimately, 6 studies met all eligibility criteria and were included in the review (Figure 2).

Due to substantial methodological and clinical heterogeneity among included studies, a quantitative meta-analysis was not performed, and findings were synthesized narratively. Only studies of moderate to high methodological quality were included in the systematic review.

**Table 1:** Methodological quality evaluation of the included studies.

No.	Author/ Year	Clearness of Stated Aim (0–2)	Sample Selection				Comparability		Outcomes		NOS Total Score (Quality Assessment) (0–16)
			Sample Representativeness (0–2)	Sample Size (0–2)	Inclusion Criteria (0–2)	Exclusion Criteria (0–2)	Allocation of Participants into Homogeneous Groups (0–1)	Number and Characteristics of Study Dropouts (0–1)	Assessment of the Outcome (0–2)	Statistical Tests (0–2)	
1.	Filatava EJ et al., 2023 [24]	2	2	2	2	2	2	0	2	2	14
2.	Dinleyici M et al., 2024 [21]	2	2	2	2	0	2	0	2	2	12
3.	Parul Singh et al., 2023 [25]	1	2	2	2	2	2	2	2	2	16
4.	Andrea C. Masi et al., 2024 [26]	2	2	2	1	0	2	0	2	2	13
5.	Sara Shama et al., 2024 [27]	2	2	2	2	2	2	2	2	2	16
6.	Schulkers Escalante K et al., 2025 [28]	2	2	2	2	0	2	0	2	2	12

### 3.2. Study Characteristics

A comprehensive review of the literature from the past five years identified six studies that met the inclusion criteria (Table 2). Collectively, these studies encompassed 318 preterm newborns of whom 287 were VLBW and their mothers, with a total of 1282 breast milk samples analyzed. Among the six studies, one was a recently published pilot investigation that nonetheless fulfilled the eligibility criteria [25].

**Table 2:** Summary of recent studies investigating the human milk microbiota in preterm VLBW infants. Characteristics of included studies.

Author/Year	Country	Study Aim	Study Population, Samples, Birth Weight/Gestation Age
Sara Shama, Michelle R. Asbury, Alex Kiss et al., 2024 [27]	Canada	To investigate the microbiota in preterm mother’s milk and the recipient VLBW infant gut and to establish whether these relationships are modified by postnatal period.	Randomized clinical trial; n = 94 mother–infant dyads; 422 milk–stool pairs. The median BW and GA of infants were 850 (730–1047) g and 27.4 (25.7–29.1) weeks.
Andrea C. Masi, Lauren C. Beck, John D. Perry et al., 2024 [26]	UK/USA	To investigate MOM microbiota of preterm NEC and gestationally matched control infants.	Prospective; n = 110 preterm newborns included in this study (NEC n = 48, control n = 62); 110 milk samples. The median BW and GA of infants in NEC group were 713 (589; 855) g and 25 (24; 27) weeks; in control—800 (640; 900) g and 26 (24; 27) weeks.
Parul Singh, Noora Al Mohannadi, Selvasankar Murugesan et al., 2023 [25]	Qatar, UK, USA, Thailand et. Cetera	To investigate MOM microbiota of preterm infants. To compare these samples across different lactation stages (colostrum, transitional and mature milk).	Case–control study; term n = 30, preterm n = 18 Two samples were collected from each woman: 0–3 days postpartum (colostrum); 7–15 days postpartum (transitional milk); 2 months postpartum (mature milk); 381 milk samples. The median BW term (≥37 weeks)—3070 (2960–3300) g, preterm (<37 weeks)—2260 (1980–2440) g.
Filatava EJ, Liu Z, Xie J et al., 2023 [24]	USA/China	To investigate MOM microbiota in mothers of VLBW preterm newborns change over the course of lactation.	Prospective; n = 72 infants and n = 65 mothers due to seven twin births; 341 enteral nutrition samples (238 MOM, 30 PDHM, and 73 formula). ≤34 weeks of gestation, the median GA 29.5 ± 2.4 weeks.
Schulkers Escalante K, Bai-Tong SS, Allard SM et al., 2025 [28]	USA	To investigate how the transition from tube to oral/breastfeeding impacts the preterm infants’ oral and gut microbiome and metabolome.	Prospective/a pilot study. n = 11 mother-infant dyads; 39 stool, 44 saliva, and 43 milk samples over 4 timepoints. The median GA 27.9 (23.4–32.2) weeks.
Dinleyici M, Pérez-Brocal V, Arslanoglu S et al., 2024 [21]	Turkey, Spain, Belgium	To compare transient and mature HM virome compositions, and also possible changes related to the mode of delivery, gestational age, and weight for gestational age.	Prospective; n = 44 mothers, n = 13 preterm; 88 human milk samples. The median BW 2255 (1200–2700) g and GA 35 (32–37) weeks.

### 3.3. Very-Low-Birth-Weight Preterm Newborns’ Mom Microbiota

Across the included studies, the MOM microbiome of preterm infants is characterized by a conserved core dominated by *Staphylococcus*, *Streptococcus*, and *Enterococcus*, as consistently reported by Shama et al., Masi et al. and Filatava EJ et al. [24,26,27]. Additional genera such as *Corynebacterium*, *Veillonella*, and *Acinetobacter* were also frequently detected [26,27,28]. In addition to bacterial communities, one study reported the presence of a milk virome, dominated by bacteriophages and including members of the *Herpesviridae* family [21] (Table 3).

### 3.4. Longitudinal Changes in the Microbiota of Mothers’ Own Milk in VLBW Preterm Infants

Microbial composition varied according to gestational age, with preterm milk showing a higher relative abundance of opportunistic and environmental taxa, including *Pseudomonas* and *Acinetobacter* [25]. Differences between preterm and term or later-stage milk were also observed, with enrichment of *Veillonella* and *Lactobacillus* in more mature samples [25] (Table 4).

Longitudinal changes across lactation stages were consistently reported. Early milk was characterized by lower diversity and dominance of *Staphylococcus*, whereas mature milk exhibited increased diversity and shifts in microbial composition [25,27]. Specifically, a decrease in *Clostridium sensu stricto* and changes in *Haemophilus* abundance over time were described [27].

Environmental influences, particularly in clinical and neonatal intensive care settings, were evident, with detection of hospital-associated taxa such as *Pseudomonas* and *Acinetobacter* [24,26].

Finally, associations between human milk microbiota and the infant microbiome were identified, with positive correlations observed for specific taxa such as *Acinetobacter*, suggesting a potential role of human milk in early microbial colonization of the infant gut [26].

### 3.5. Links Between Mothers’ Own Milk Microbiota and Clinical Outcomes in Very-Low-Birth-Weight Infants

The reviewed literature revealed just one study reporting a possible association between the microbiota of mothers’ milk and NEC [26] in VLBW preterm infants, while investigations of other neonatal health outcomes were absent. However, no significant differences in MOM microbiota composition or bacterial load were found between infants with NEC and healthy controls within an exclusively preterm neonatal intensive care unit (NICU) cohort [26]. Recent research continues to emphasize the importance of human milk bioactive components and gut microbial development in reducing NEC risk and promoting overall health in preterm populations.

## 4. Discussion

The influence of nutritional management on microbiota development and clinical outcomes in very preterm neonates remains incompletely understood [22]. To date, relatively few studies have investigated the MOM microbiota of mothers delivering VLBW infants using sequencing-based approaches [26,27].

The MOM microbiota in VLBW preterm mothers shows temporal and compositional changes influenced by lactation stage, feeding, and maternal factors [24,26,27,28]. Preterm milk demonstrates higher species richness and is enriched in *Staphylococcus haemolyticus*, *Propionibacterium acnes*, and unclassified Corynebacterium, whereas mature milk favors colonization by Veillonella and Lactobacillus, reflecting progressive microbial maturation over the course of lactation [25,29]. Longitudinal studies highlight that microbial diversity and composition are not static; direct breastfeeding enhances the transfer of key commensals including Staphylococcus, Veillonella, *Streptococcus*, and Haemophilus to the infant gut, with dose-dependent relationships most pronounced during the first postnatal month under predominant mother’s milk feeding [20,24]. These findings underscore the role of maternal milk as a primary vector for early gut colonization and the establishment of a foundational microbiome in VLBW infants [30].

Feeding type further shapes neonatal microbial exposures, with MOM, PDHM pasteurized donor human milk (PDHM), and formula each exhibiting unique bacterial profiles [26]. MOM is dominated by Staphylococcus and Pseudomonas, PDHM displays more balanced proportions of Staphylococcus, Pseudomonas, and *Streptococcus*, while formula is enriched in Lactococcus and *Streptococcus*, illustrating how both maternal and environmental factors modulate microbial delivery [24,31]. The identification of distinct MOM “lactotypes” suggests that maternal determinants may influence the functional potential of milk microbiota, potentially affecting immune maturation, gut barrier development, and metabolic programming in preterm infants [26]. These data collectively support the concept that preterm MOM is a dynamic, biologically active milieu that mediates both microbial and immunological shaping of the neonatal gut.

Although direct associations between MOM microbiota and NEC incidence remain limited, integrative analyses indicate that microbial and bioactive components of milk work synergistically to reduce gut inflammation and promote resilience in the preterm intestine [28]. The observed associations between the quantity of bacteria consumed and the establishment of the infant gut microbiota, together with metabolomic evidence linking milk composition to key nutrient pathways, highlight the mechanistic plausibility of MOM-mediated protection against inflammatory disorders [24,25]. Although these studies have enhanced our understanding of early-life microbiome development and its influence on health outcomes, knowledge regarding the microbiota composition of preterm infants remains limited [21]. Future research should investigate the influence of host genetic factors and immune function on the milk microbiota. In addition, elucidating interactions among bacterial species, as well as relationships between bacteria and other microorganisms such as fungi and viruses, will be essential for clarifying the factors that shape microbial communities in human milk [32]. Addressing these gaps will be critical to elucidate how MOM microbiota shapes gut colonization, supports immune development, and optimizes health trajectories in VLBW preterm infants.

No studies to date have analyzed the fungal or archaeal components of breast milk microbiome in mothers of premature infants.

Collectively, these findings suggest that the microbiota of MOM evolves over time and is influenced by maternal and environmental factors. Moreover, distinct microbial community structures referred to as “lactotypes” have been identified within MOM [24], each characterized by unique bacterial profiles and maternal determinants. These observations underscore the dynamic nature of the breast milk microbiome and emphasize the importance of additional studies aimed at clarifying how specific lactotypes and temporal shifts in milk microbiota contribute to gut colonization, immune maturation, and overall health outcomes in preterm and VLBW infants.

## 5. Conclusions

MOM in VLBW preterm infants contains a dynamic and distinct microbiota that evolves throughout lactation and plays an important role in early neonatal microbial colonization. Across the reviewed studies, a relatively conserved core microbiome was identified, predominantly composed of *Staphylococcus*, *Streptococcus*, *Enterococcus*, and other frequently detected genera such as *Corynebacterium*, *Veillonella*, and *Acinetobacter*. These microbial communities vary according to gestational age, stage of lactation, feeding practices, and environmental exposures, particularly within the neonatal intensive care setting.

Preterm milk showed a higher abundance of opportunistic and hospital-associated bacteria, whereas mature milk had greater diversity and more beneficial genera such as *Veillonella* and *Lactobacillus*, indicating microbial maturation over time. Mothers’ own milk was also closely associated with infant gut microbiota, supporting its important role in gut colonization and immune development in VLBW infants. Although evidence linking MOM microbiota to outcomes such as NEC remains limited, microbial transfer through human milk may support intestinal protection, immune regulation, and neonatal resilience. The presence of viral components, especially bacteriophages, further highlights the complexity of the milk microbiome beyond bacteria. Overall, MOM should be considered not only as optimal nutrition but also as a biologically active source of microbial and immunological factors essential for the development of VLBW preterm infants. Further large-scale longitudinal studies using advanced multi-omic approaches are needed to better define the functional significance of specific microbial patterns, lactotypes, and non-bacterial components, and to clarify their relationship with short- and long-term health outcomes in VLBW preterm newborns.

## 6. Limitations

Research on bacterial phyla in preterm human milk remains limited, with relatively few studies exploring this area. For example, there has not been much research conducted on this topic, and there is a lack of scientific clinical trials. Existing studies often rely on small sample sizes, reducing the generalizability of findings and limiting the detection of subtle associations with neonatal outcomes. Additionally, the biomolecular techniques used have been narrow in scope, preventing a comprehensive characterization of the milk microbiota. Future research should incorporate larger cohorts and advanced multi-omic approaches to better understand the complexity and clinical relevance of the preterm milk microbiome, including the roles of non-bacterial components such as fungi, archaea, and viruses.

## Figures and Tables

**Table 3:** Diversity of the MOM microbiome of VLBW newborns mothers.

Author	Human Milk Maturity	MOM Microbiome
Sara Shama, Michelle R. Asbury, Alex Kiss et al. [27]	preterm	*Enterobacteriaceae*, *Staphylococcus*, *Enterococcus*, *Streptococcus*, *Veillonella*, *Clostridium sensu stricto*, *Acinetobacter*, *Corynebacterium*, *Pseudomonas*, *Haemophilus*, *Finegoldia* and *Bifidobacterium*.
Andrea C. Masi, Lauren C. Beck, John D. Perry et al. [26]	preterm	Preterm samples. The 10 most abundant bacteria in rank order were *Staphylococcus* (57.1%), *Acinetobacter* (13.9%), *Enterobacter* (7%), *Pseudomonas* (5.7%), *Enterococcus* (5.4%), *Corynebacterium* (2.8%), *Cutibacterium* (1.2%), *Finegoldia* (0.9%), *Streptococcus* (0.9%), and *Bifidobacterium* (0.8%).
Parul Singh, Noora Al Mohannadi, Selvasankar Murugesan et al. [25]	term/preterm	Preterm samples—*Staphylococcus haemolyticus*, *Propionibacterium acnes*, unclassified *Corynebacterium* species. Term samples were enriched in *Staphylococcus epidermidis*, unclassified OD1, and unclassified *Veillonella* among others.
Filatava EJ, Liu Z, Xie J et al. [24]	preterm	*Staphylococcus*, *Lactococcus*, *Streptococcus*, *Pseudomonas*, *Propionibacterium*, *Enterococcus*, *Corynebacterium*, *Acinetobacter*, *Prevotella*, *Finegoldia*, unclassified *Enterobacter*ia, *Lactobacillus*, *Stenotrophomonas*, *Klebsiella*, *Actinomyces* and *Anaerococcus*.
Schulkers Escalante K, Bai-Tong SS, Allard SM et al. [28]	preterm	The most differentially represented top 20 taxa were assigned to *Enterobacter*, *Streptococcus*, *Klebsiella*, *Veillonella*, *Rothia* and *Mesorhizobium*.
Dinleyici M, Pérez-Brocal V, Arslanoglu S et al. [21]	term/preterm	*Caudovirales* (74.8%), particularly families *Siphoviridae* (34.6%), *Podoviridae* (24.6%), and *Myoviridae* (14.4%), with species *Staphylococcus* phage St 134 (1.9%), *Streptococcus* phage IPP62 (1.2%), *Staphylococcus* phage Andhra (1.2%), *Acinetobacter* virus 133 (1.1%), *Staphylococcus* virus SEP (1%), *Enterococcus* phage EFC-1 (0.91%), *Streptococcus* phage YMC-2011 (0.85%), *Staphylococcus* virus IPLAC1C (0.7%), *Staphylococcus* virus Sextaec (0.67%), and *Clostridium* phage vB_CpeS CP51 18 (0.54%) being the most abundant ones.

**Table 4:** Longitudinal changes in the milk microbiome of preterm mothers during lactation.

Author	Lactation-Associated Changes
Sara Shama, Michelle R. Asbury, Alex Kiss et al. [27]	Feeding an infant at the breast increased *Veillonella*, *Streptococcus*, *Haemophilus*, and *Clostridium sensu stricto*.
Parul Singh, Noora Al Mohannadi, Selvasankar Murugesan et al. [25]	Mature milk samples were significantly enriched in *Veillonella* and Lactobacillus compared to colostrum.
Filatava EJ, Liu Z, Xie J et al. [24]	Pseudomonas, unclassified *Enterobacteria*, *Klebsiella*, *Lactococcus*, *Streptococcus*, *Acinetobacter* and Rothia *increased*, while *Staphylococcus*, *Finegoldia* and *Peptoniphilus* decreased over time.

## Data Availability

No new data were created or analyzed in this study.

## Supplementary Materials

The following supporting information can be downloaded at: https://www.mdpi.com/article/10.3390/children13060790/s1, Table S1: PRISMA 2020 Checklist.
